# Using GPS technology to (re)-examine operational definitions of ‘neighbourhood’ in place-based health research

**DOI:** 10.1186/1476-072X-11-22

**Published:** 2012-06-27

**Authors:** Bryan J Boruff, Andrea Nathan, Sandra Nijënstein

**Affiliations:** 1 School of Earth and Environment, University of Western Australia, 35 Stirling Hwy, Crawley, WA, 6009, Australia; 2 Centre for the Built Environment and Health, University of Western Australia, 35 Stirling Hwy, Crawley, WA, 6009, Australia; 3 School of Innovation Sciences, Eindhoven University of Technology, P.O. Box 513, Pav. B08.a, 5600 MB, Eindhoven, Netherlands

**Keywords:** Built environment, Physical activity, Neighbourhood effects, Older adults, GPS, GIS

## Abstract

**Background:**

Inconsistencies in research findings on the impact of the built environment on walking across the life course may be methodologically driven. Commonly used methods to define ‘neighbourhood’, from which built environment variables are measured, may not accurately represent the spatial extent to which the behaviour in question occurs. This paper aims to provide new methods for spatially defining ‘neighbourhood’ based on how people use their surrounding environment.

**Results:**

Informed by Global Positioning Systems (GPS) tracking data, several alternative neighbourhood delineation techniques were examined (i.e., variable width, convex hull and standard deviation buffers). Compared with traditionally used buffers (i.e., circular and polygon network), differences were found in built environment characteristics within the newly created ‘neighbourhoods’. Model fit statistics indicated that exposure measures derived from alternative buffering techniques provided a better fit when examining the relationship between land-use and walking for transport or leisure.

**Conclusions:**

This research identifies how changes in the spatial extent from which built environment measures are derived may influence walking behaviour. Buffer size and orientation influences the relationship between built environment measures and walking for leisure in older adults. The use of GPS data proved suitable for re-examining operational definitions of neighbourhood.

## Background

The influence of the built environment on healthy behaviours such as walking has received significant attention in the past decade [1,2]. Scholars have identified the composition of a person’s surrounding environment (or ‘neighbourhood’) to be important in their propensity to walk [1,3-5]. For older adults – the fastest growing population group in society – a small but growing body of evidence highlights some significant associations between environmental attributes and walking [6-9]. For example, walkability indices composed of street connectivity, residential density, and land-use mix measures, show higher walkability to be positively related to older adults’ leisure walking [10,11] and transport walking [11-14]. Others report the presence and proximity to retail or commercial destinations [15,16] and parks and open green space [17,18] to be significant correlates of walking. However, most research reports inconsistent findings, making it difficult to understand environmental influences on walking for older adults [19]. It is possible that such inconsistencies in research findings are methodologically driven. Across the board, variation arises in the operationalization and spatial extent of what is considered ‘neighbourhood’ [20].

Methods typically used to define neighbourhood include: predefined spatial units; circular buffers; and polygon-based road network buffers [21]. But predefined spatial units, such as census tracts or other political units, do not necessarily represent the area in which a person walks and presents issues of adjacency when a person lives on the edge of the predefined area. Alternatively, circular buffers, which define a neighbourhood as the area surrounding an individual’s home and are often measured at distances ranging from 400 m to 1600 m to reflect a five to 20 minute walk, have received wide acceptance [5,22-26]. However, researchers have argued that the spatial footprint of walking is influenced by the road network, therefore polygon-based road network buffers are thought to provide a more accurate representation of neighbourhood. Furthermore, Oliver et al. [21] introduced a buffered line-based network approach, whereby only spatial phenomena alongside the road network is taken into account; that is, the immediate lands to which people are exposed to along the walking route. The authors concluded that this new measure was more sensitive than circular buffers and polygon-based network buffers, resulting in a greater association between built environment measures and walking. These results highlight the opportunity to re-examine traditional neighbourhood delineation techniques in an attempt to produce more appropriate measures of the built environment.

The use of buffers (circular or polygon network) to define the spatial extent for which measures of the built environment are derived has been the target of increasing criticism [27-29]. Spatial phenomena such as transport infrastructure, major roads and commercial establishments influence the orientation of the neighbourhood to which people are exposed [27]. Furthermore, human activity has both a temporal and spatial component and by limiting place-based research to a single residence and predefined area at one point in time, the ‘contextual terrain’ that influences health is lost [28]. Empirically speaking, Spielman and Yoo [29] argued that inorganic boundaries imposed by health researchers underestimate the influence of the environment on health by discounting individual-level spatial variability. Whilst Spielman and Yoo [29] call for a theoretical shift away from fixed neighbourhoods, others take a more pragmatic approach identifying that alternative spatial definitions of neighbourhoods exist (standard deviation ellipse, minimum convex polygon and kernel density estimations) and may provide a better measure of individual environments [28,30]. What is common in all of these critiques however, is the identification of Global Positioning Systems (GPS) technology as an opportunity for examining the spatial interaction between humans and their environment, ultimately providing the prospect for a re-examination of the spatial definition of ‘neighbourhood’.

Studies using GPS technology to measure location-based physical activity are emerging in the health literature. For example, Badland et al. [31] recently compared exposure to different built environment features based on participant route and mode of transportation for commuting to work (shortest route, public transport, walking, and personal vehicle). Troped and colleagues [32] examined the relationship between moderate-to-vigorous physical activity (MVPA), conducted within 50 m and 1 km buffers of participant’s home and place of work, and several measures of the built environment (connectivity, population density, land-use mix and a vegetation index). In addition to examining physical activity patterns in adults, GPS technology is finding application in tracking the movement of children for much the same purpose. GPS derived data has been used to examine seasonal and daily patterns of physical activity in children [33], as well as time and distribution of MVPA [34]. Several studies have gone further, examining the relationship between type and vigour of children’s physical activity and the location in which it occurs [35-37].

Possibly the first study of its kind, Zenk et al. [30] utilised GPS track data to create two new delineation techniques - a standard deviation ellipse and GPS point buffering approach – and subsequently examined relationships between built environment measures (fast food outlet density and supermarket availability) and dietary intake. Findings suggested that ‘activity space’ may have a greater influence on behaviour than the traditional radial or polygon network based neighbourhoods often utilised in spatial health research. But while this exploratory work highlights the possibilities for using GPS data to redefine our understanding of neighbourhood buffers, one limitation was its lack of an explicit comparison with the more traditional buffering techniques. Therefore, the present pilot study sought to fill this void by using GPS derived data to not only create new buffering approaches, but then to compare them with traditional approaches when examining relationships between built environment measures and walking for transport and leisure. Specifically, this paper aims to:

Create alternative neighbourhood buffers, based on objective GPS walking data, which better represent the spatial extent in which older adults actually walk;

Explore the differences between the new neighbourhood buffers and standard buffers commonly used in the literature (i.e., circular, polygon network, line-based network buffers) for built environment measures; and

Examine the relationship between land-use exposure and self-reported walking in older adults for each neighbourhood buffer.

## Methods

### Participants

The Active Living Study – a mixed-method, cross-sectional study conducted in 2009 – investigated active living among retirement village residents and the influence of village and neighbourhood environments. The exploratory qualitative phase informed the development of a quantitative survey instrument, which was administered to 325 residents in 32 retirement villages across Perth, Australia. A convenience sub-sample of residents from seven of the 32 study villages were approached to take part in the GPS component (n = 74). Additional information sheets were provided, and written consent received from 41 residents (55%). Ethics approval was granted by The University of Western Australia Human Research Ethics Committee (RA/4/1/2151).

### Data collection

Study participants met with researchers on a pre-arranged day and time within the retirement village to complete a questionnaire and receive equipment. Among other items, the questionnaire included demographics (gender, age, highest level of education attained, marital status), self-reported height and weight (used to calculate body mass index [BMI]), and previously validated and reliable measures of physical activity [38,39] and physical functioning [40]. Actigraph GT1M accelerometers, initialized to collect data in one minute epochs, were distributed to participants on an elastic belt along with instructions to wear the device around the waist, on the right side, during waking hours (except during water-based activities), for seven days. An accelerometer diary was provided to encourage compliance and monitor daily wear time.

The GPS sub-sample received a GlobalSat DG-100 data logger on an additional elastic belt, and were required to wear the device at the same time as their accelerometer, on the left side of the body. The epoch length was 15 seconds. Additional verbal and written instructions were given, highlighting the need to recharge the GPS unit each night. Similar to the accelerometer diary, a GPS diary to encourage compliance, monitor daily wear time, and remind participants to recharge the device was distributed.

### GPS data treatment

Approximately 126 MB of GPS data and 10 MB of accelerometer data were collected for the 41 sub-sample participants. These data were processed using the Physical Activity Location Measurement System (PALMS) currently under development at the University of California, San Diego School of Medicine and California Institute for Telecommunications and Information Technology [41]. Briefly, PALMS provides the ability to align physical activity measurement sensors such as GPS and accelerometer data based on time of capture, and define the start and end points of trips, calculates trip speed, distance and duration, and classifies trips based on transport mode (i.e., vehicle, bicycle, pedestrian, or stationary).

To classify GPS data into trip type, PALMS first filters invalid values based on speed, change in elevation, change in distance between satellite fixes and period of time for which loss of signal is declared [42]. Next, PALMS identifies if a GPS point was collected indoors or outdoors based on the number of satellites detected during each fix and the satellite signal to noise ratio. PALMS then uses a state-based method classifying GPS points as stationary, moving, or paused requiring that a set of criteria are met (i.e. minimum distance travelled in one minute) before a change in state can occur (i.e. stationary to moving). Trips are then classified as vehicle, bicycle or pedestrian based on speed cutoff values. PALMS documentation suggests that trip parameters should be based on the ‘population under study’ which for our sample required several iterations to identify the most appropriate thresholds. To classify trips in our study, speeds less than 7 kilometres per hour indicated walking, 8 – 19 indicated bicycling and greater than 20 indicated vehicle travel.

All trips classified as ‘pedestrian’ by PALMS were then visually assessed by first overlaying the GPS points over high-resolution aerial photography in ArcMap 9.3. Average speed (as calculated by PALMS) was visually inspected to identify anomalies in the data where speed of movement did not match the associated terrain (i.e. were speeds associated with automobile travel were found within a park). In some cases, a visual examination of increases or decreases in speed, and location of stationary points resulted in manually splitting an identified trip into two or more trips (i.e. automobile and walking). Assessment criteria for identifying walking trips from GPS data as outlined by Cho et al. [43] were also applied. Criterion included: a maximum time gap between points of three minutes to identify the start and end of trips; trips of at least five minutes duration; mean trip speed no more than eight kilometres per hour; and at least 30 meters of displacement between trip start and end points. This led to a final data set focussing exclusively on walking trips.

### Land-use exposure

**Figure 1:**
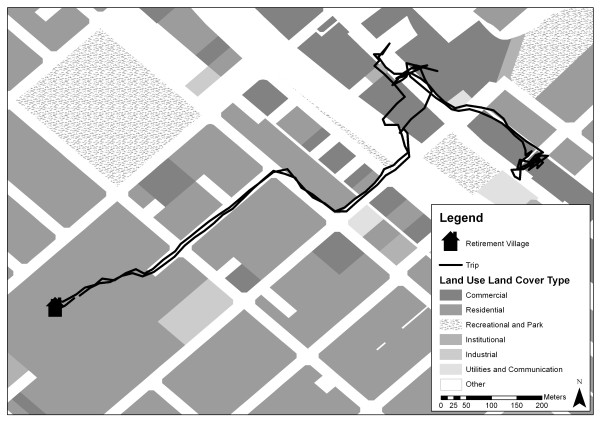
Example of a GPS track for one walking trip and land-use exposure.

The original GPS points making up each identified walking trip were first converted into a continuous line using Hawth’s Tools (Figure 1). Hawth’s Tools is a free ArcGIS extension which provides ecologically focused functions not easily accomplished in ESRI software [44]. Of importance to this study are the ‘animal movement’ tools, one of which allows users to convert a series of points to a single path (or line). In order to measure land-use exposure along the immediate walking route, trips were buffered by 50 meters (on either side), as proposed by Oliver et al. [21].

Percentage of land-use type participants were exposed to along the walking trip was calculated. To this end, parcel data acquired from the State Government’s Department of Planning and land-use information from the Value Generals Office (VGO) were spatially combined. The VGO data consisted of point locations representing over 1200 categories of rateable (or taxable) features identified by the state. The point features were spatially joined with their corresponding cadastral parcel and reclassified to 16 general categories, then further aggregated to seven land-use types, modelled after those used by Oliver et al. [21]. The result was a cadastral based land-use classification where each cadastral parcels was assigned one of the following land-use categories: c*ommercial land* included retail land, offices/businesses, and service industries; *institutional land* included health/welfare, community services and schools; *recreational and park land* included entertainment/recreational and cultural land, public open space, and sporting infrastructure; *industrial land* included storage, distribution, manufacturing, processing, and fabrication; *residential land* included all dwellings and homes; *utilities/communications* such as water, electrical and communication infrastructure; and *other land* included streets, and vacant, primary/rural and unclassified land (i.e., areas lacking any land-use data).

In addition to the parcel based land-use classification, geocoded point location data from the Australian Yellow Pages (Sensis Pty. Ltd.) provided supplemental information concerning the specific types of ‘destinations’ participants were exposed to. Using SENSIS data and high resolution aerial photography, walking trips were further examined visually by the researchers, to determine the destination types that participants walked to or were exposed to along a route. This approach was used to validate previous research reporting high levels of individual exposure to recreational, institutional and commercial establishments whilst engaging in physical activity [45,46]. Finally, the destination point data was used in the development of new buffering techniques discussed in the next section.

### New buffer development

Four new geographically-informed neighbourhood buffers, which better reflect where residents actually walk, were developed: a Variable Width buffer; *recreationalinstitutional* and *commercial* (RIC) line buffer; RIC polygon buffer; and RIC ellipse buffer. In short, variable width buffers allow the extent of a buffer to be varied spatially based on ancillary information, whilst the RIC buffers use point locations representing *recreationalinstitutional* and *commercial* locations to constrain the shape of the buffer. The new buffers were based on land-use and destination exposure results from the GPS data derived walking trips. This information was then used as parameters for the development of a suite of new buffering techniques which are applied to the entire population of study participants. As such, buffers were created for all 32 retirement villages in the study at 1000 m distance, as has been done in other studies among older adults [47-49]. A visual examination of walking trips identified that the furthest (Euclidean) distance any one participant walked from their home was approximately 1200 metres, further justifying the choice to constrain buffer sizes to 1000 metres. Buffer sizes were explored for other distances but because results were similar, only 1000 m buffers are presented in this manuscript. Though the buffers were created based on land-use exposure data from a sub-sample of n = 7 villages, no significant differences were found for percentage land-use type within each buffer when compared with all villages studied (i.e., n = 32).

Variable Width buffers, used primarily in the field of ecology, were initially developed to identify the land area on either side of a stream to be set aside or repaired to maintain ecosystem integrity. They are generally based on stream size, presence of fish, land-use encroaching the stream or all of the above [50,51]. Based on this principle, and using the mean percentage land-use exposure on GPS walking trips, weights were developed to reflect land use types that, on average, participants were more exposed to during their walking trips. Weights represent one minus the mean percent exposure to each land-use type for all walking trips (Table 1). For example, participants were only exposed to 1% of *industrial* land-use along walking trips, therefore moving across this land-use type incurred a weight of 0.99 significantly constraining the amount of *industrial* land-use within a variable width neighbourhood buffer.

**Table 1:** Land-use type, mean percent exposure and weights

**Land-use type**	**Mean % Exposure¹**	**Land-use weight**
Residential	43	0.57
Other	36	0.64
Commercial	12	0.88
Recreational and park	7	0.93
Institutional	2	0.98
Industrial	1	0.99
Utilities/Communications	1	0.99

¹Mean % exposure equals 102 due to rounding.

**Figure 2:**
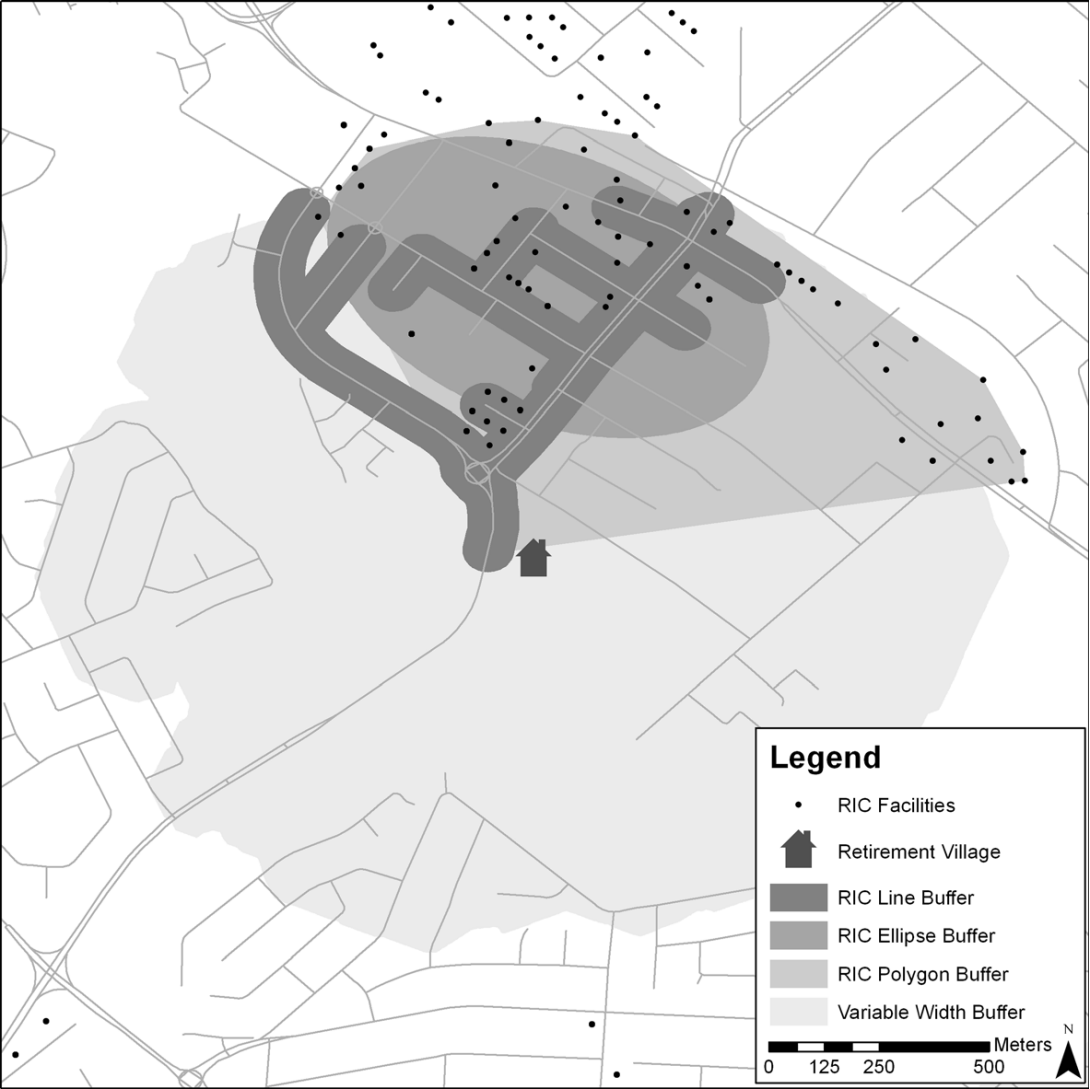
Example of the Spatial Extent of a RIC Line Buffer, RIC Ellipse Buffer, RIC Polygon Buffer and Variable Width Buffer.

A land-use grid (5 m x 5 m cell size) was developed for the study area and weights (Table 1) were ascribed to each land-use type to develop a cost surface. In computational terms, as a participant moved across a grid cell (land-use type), weights were accumulated (i.e., travel cost). As the *residential* land-use type incurred the lowest weight, the travel cost of moving across this type of land-use was calculated for a distance of 1000 m from the centroid of a retirement village (i.e., the maximum distance any one participant could travel from their home as the crow flies). This number represented the highest cost a participant could accrue for moving across the landscape if only exposed to *residential* land and was used to constrain the distance a participant could travel across the land-use grid. The travel cost of moving a certain distance from the centroid of each retirement village was then calculated using the ArcGIS Spatial Analyst Cost Distance tool using the weights derived for each land-use type. The resulting neighbourhood buffer represents a zone showing the maximum distance a person could travel from a retirement village before they accumulated an equivalent cost of travelling 1 km over residential land-use only (Figure 2).

The remaining three buffers were based on the types of destinations that participants walked to or were exposed to along the GPS walking trips. These were identified by visually examining the GPS walking trips in relation to the destination point data derived from the Yellow Pages listings. Visual examination of the walking trips revealed that participants were primarily exposed to *recreational and parkinstitutional*, and *commercial* (RIC) facilities. Similar findings have been reported in several studies [45,46]. Therefore, the point locations of RIC facilities were used to develop these buffers (Figure 2). For the RIC line buffer, the shortest routes to all RIC facilities within 1000 metres (road network meters) of the retirement village were buffered at 50 m [21]. The routes were created using ArcGIS Network Analyst Closest Facility tool. RIC polygon buffers were created with the Hawth’s Convex Hull tool. The smallest convex was generated including all RIC points within 1000 metres (radial meters) of each retirement village.

Finally, all RIC facilities within a 1000 m (radial buffer) of each retirement village were used to develop the RIC ellipse buffers. This was done with the ArcGIS Spatial Statistics Standard Deviational Ellipse tool. Note that the choice of using the standard deviation (SD = 1) to create the ellipses highlights the directional trend of RIC facilities within the data but does not include all input points including the retirement village. This approach varies from that used by Zenk et al., [30] who used only GPS track points to calculate standard deviation ellipses. Our approach only uses the RIC facilities to constrain the ellipse buffers so the approach could be extended to the entire study sample (n = 325). Whilst this approach does not always capture land-use exposure from a starting point (i.e. the retirement village), it does provide an accurate delineation of the geographic area containing facilities a participant may be exposed to.

### Standard buffer development

**Figure 3:**
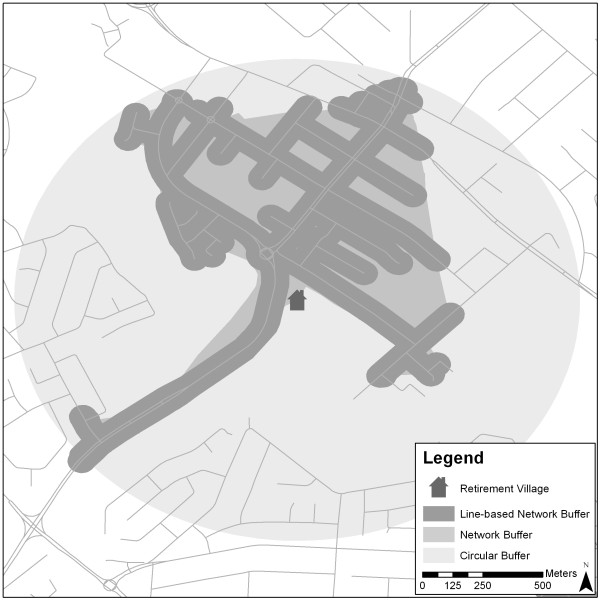
Example of the Spatial Extent of Line-based Network Buffers, Polygon Network Buffers and Circular Buffers Used in the Study.

In order to compare the new buffers with those commonly used in the literature, three standard buffers were developed. Similar to the new buffers, standard buffers were created for all 32 retirement villages in the study at 1000 m distance. Percentage land-use type within each buffer was again calculated. Standard radial and polygon network buffers were calculated in ArcMap 9.3. Using the road segments identified from the polygon network buffer, the line-based network buffer was created by buffering either side of the road centerline by 50 m. The line-based network buffer included all parcels along the road whilst excluded those further away. Figure 3 provides an example of these three buffers.

### Statistical analyses

Demographic characteristics of the sample were compared using cross-tabulation and chi-square statistics or independent t-tests for continuous variables. In order to explore the differences in percentage land-use type between the four new buffers (variable width, RIC polygon, RIC ellipse, and RIC line) and the three standard buffers (circular, polygon network, and line-based network), Spearman’s rho correlation coefficients were estimated. To examine relationships between percentage land-use type in each buffer and walking for the total study sample (i.e., n = 325), single questionnaire items on walking for leisure and walking for transport were dichotomized (i.e., some vs. none), and logistic regression models fitted with Generalised Estimating Equations using an exchangeable correlation matrix to adjust for clustering. All models adjusted for gender, age, highest education level completed, marital status, BMI, and physical functioning score. Analyses were conducted using PASW Statistics 18.

## Results

### GPS walking trips

Initially, PALMS detected 1632 trips, of which 544 were classified as ‘pedestrian’. Walking trips within the retirement village were then excluded resulting in 80 walking trips for 19 participants (Table 2). Trips per participant ranged from one to 17 with a mean of 4.16 walking trips per participant. Average walking trip duration was nearly 26 minutes with a mean distance of 1.44 kilometres.

**Table 2:** Characteristics of GPS walking trips (n = 80)

**Characteristic**	**Mean**	**SD**	**Minimum**	**Maximum**
Trips per participant	4.16	3.83	1.00	17.00
Trip duration (minutes)	25.99	19.45	5.75	137.25
Trip length (kilometres)	1.44	0.96	0.22	5.66
Trip speed (kilometres per hour)	3.74	1.05	1.59	7.44

### Participant characteristics

Demographic characteristics are presented in Table 3. For the total sample (n = 325), 68% were female, 11% completed a bachelor’s degree or higher, and 53% were married. Participant mean age was nearly 77 years and physical functioning was fairly high across the sample. Compared with the total sample, individuals approached to participate in the GPS component of the study (n = 74) did not differ significantly in any demographic factors (i.e., gender, age, education, marital status, physical functioning, and BMI) from those not invited to participate. GPS sub-sample participants (n = 41) were significantly younger (p = 0.024), had higher physical functioning (p = 0.024), and higher BMI scores (p = 0.022) than the overall sample, while sub-sample participants who had walking trips outside of the retirement village (n = 19) were significant younger (p = 0.015), and appeared to have higher physical functioning than the total sample, though this only approached statistical significance (p = 0.066).

**Table 3:** Description of characteristics

**Characteristic**	**Total sample (n = 325)**	**GPS study invite (n = 74)**	**p**	**GPS participant (n = 41)**	**p**	**GPS walking trips (n = 19)**	**p**
Gender (%)			0.908		0.421		0.164
Male	32.31	31.08		39.02		50.00	
Female	67.69	68.92		60.98		50.00	
Highest education level (%)			0.802		0.746		0.140
Secondary or less	47.69	47.30		43.90		27.78	
Trade/certificate	40.92	43.24		46.34		50.00	
Bachelor or higher	11.39	9.46		9.76		22.22	
Marital status (%)			0.929		0.947		0.148
Not married	47.08	54.05		48.78		72.22	
Married	52.92	45.95		51.22		27.78	
Age (mean years/SD)	76.82 (7.43)	75.97 (7.02)	0.263	74.39 (7.16)	0.024	72.72 (7.80)	0.015
Physical functioning (mean score/SD)	80.84 (16.05)	82.04 (14.78)	0.466	85.65 (13.65)	0.024	87.80 (13.93)	0.066
BMI (mean score/SD)	25.02 (4.51)	25.30 (4.57)	0.540	26.52 (4.63)	0.022	26.47 (4.70)	0.159

Note that all evaluated differences were compared with the total sample (n = 325).

### Comparison of buffers

The median percentage land-use exposure was calculated for each land-use type in each buffer for all 32 retirement villages in the study (Table 4). Within their neighbourhoods, participants were most exposed to *residential*, *commercial*, and *recreational and park* and least to *utilities/communications* and *industrial* land. As most participants lived in residential areas, *residential* land-use exposure was highest across all buffers, with the exception of the RIC line buffer. Exposure to *industrial, utilities/communication* and the *other* category were similar across all buffers.

**Table 4:** Variation in percentage land-use exposure by buffer (n = 32)

**Land-use type**	**Circular buffer**		**Polygon Network buffer**		**Line-based network buffer**		**Variable width buffer**		**RIC polygon buffer**		**RIC ellipse buffer**		**RIC line buffer**	
	**Med**	**IQR**	**Med**	**IQR**	**Med**	**IQR**	**Med**	**IQR**	**Med**	**IQR**	**Med**	**IQR**	**Med**	**IQR**
Commercial (%)	4.14	6.75	3.50	7.21	3.10	6.33	3.59	6.67	7.96	12.18	17.61	27.10	9.34	14.35
Institutional (%)	3.94	6.72	2.64	4.14	1.95	2.81	2.20	4.81	3.93	8.61	3.38	6.57	3.94	5.42
Recreational and park (%)	5.90	4.87	5.21	7.00	4.13	4.63	5.97	4.59	6.44	6.16	5.07	9.10	4.32	9.23
Industrial (%)	0.20	1.11	0.04	0.48	0.05	0.47	0.10	0.76	0.27	1.36	0.05	1.60	0.00	0.87
Residential (%)	39.70	15.96	47.23	17.49	42.26	14.19	42.26	14.19	41.25	16.08	32.71	25.57	33.93	16.90
Utilities/Communications (%)	0.37	0.95	0.28	0.97	0.31	1.02	0.30	0.82	0.34	0.55	0.54	1.16	0.12	0.62
Other (%)	36.51	17.16	36.55	13.27	38.55	14.81	36.07	12.67	31.74	12.04	30.69	15.07	38.57	13.50

The new variable width buffer was fairly similar to the standard buffers, however the use of commercial destinations and recreational and park facilities in creating the three RIC buffers (polygon, ellipse and line) altered the percent land-use exposure calculated for each toward *commercial* and *recreational and park*. For the RIC buffers, proportion of commercial land-use was greater than the proportion of recreational and park land-use, whereas for the standard and variable width buffers, recreational and park land-use exposure was greater. Even though institutional destinations were used in the creation of the RIC buffers, exposure remained similar across all buffers.

Table 5 presents the Spearman’s rho correlation coefficients for new and standard buffers. Percentage land-use exposure within the variable width buffer was strongly related to the standard buffers, with rho coefficients ranging from 0.60 to 0.99. Though the relationships were not as strong, the RIC polygon buffer also showed a strong relationship with the standard buffers. Medium relationships were found between the RIC ellipse buffer and the circular buffer, but the strength of relationship remained large for polygon network and line-based network buffers. The only exceptions were for percentage exposure to *institutional* land-use in the polygon network buffer and percentage exposure to *commercial* land-use in the line-based network buffer, which showed a percentage variance of 23.04% and 20.25% respectively. Finally, the RIC line buffer showed a strong relationship with both the polygon network and line-based network buffers. However, there was only 23.04% overlap between the RIC line and circular buffers for percentage exposure to *recreational and park* lands. Moreover, the percentage variance for percentage exposure to *residential* land-use was 22.09%.

**Table 5:** Correlations between new buffers and standard buffers for percentage land-use exposure (n = 32)

	**Variable width buffer**		**RIC polygon buffer**		**RIC ellipse buffer**		**RIC line buffer**	
	**rho**	**p**	**rho**	**p**	**rho**	**p**	**rho**	**p**
**Circular buffer**								
Commercial (%)	0.94	<0.001	0.55	0.002	0.46	0.010	0.55	0.001
Institutional (%)	0.92	<0.001	0.89	<0.001	0.62	<0.001	0.32	0.082
Recreational and park (%)	0.94	<0.001	0.61	<0.001	0.45	0.013	0.48	0.006
Industrial (%)	0.91	<0.001	0.88	<0.001	0.80	<0.001	0.63	<0.001
Residential (%)	0.99	<0.001	0.82	<0.001	0.36	0.051	0.47	0.008
Utilities/Communications (%)	0.89	<0.001	0.58	0.001	0.48	0.008	0.51	0.004
Other (%)	0.96	<0.001	0.65	<0.001	0.45	0.012	0.58	0.001
**Polygon Network buffer**								
Commercial (%)	0.78	<0.001	0.66	<0.001	0.51	0.004	0.95	<0.001
Institutional (%)	0.78	<0.001	0.65	<0.001	0.48	0.007	0.72	<0.001
Recreational and park (%)	0.60	<0.001	0.58	0.001	0.66	<0.001	0.83	<0.001
Industrial (%)	0.74	<0.001	0.78	<0.001	0.63	<0.001	0.87	<0.001
Residential (%)	0.70	<0.001	0.82	<0.001	0.52	0.003	0.67	<0.001
Utilities/Communications (%)	0.93	<0.001	0.67	<0.001	0.62	<0.001	0.61	<0.001
Other (%)	0.78	<0.001	0.67	<0.001	0.54	0.002	0.74	<0.001
**Line-based network buffer**								
Commercial (%)	0.76	<0.001	0.60	<0.001	0.45	0.012	0.94	<0.001
Institutional (%)	0.78	<0.001	0.69	<0.001	0.53	0.003	0.69	<0.001
Recreational and park (%)	0.64	<0.001	0.55	0.002	0.65	<0.001	0.81	<0.001
Industrial (%)	0.73	<0.001	0.78	<0.001	0.67	<0.001	0.84	<0.001
Residential (%)	0.67	<0.001	0.83	<0.001	0.52	0.003	0.63	<0.001
Utilities/Communications (%)	0.90	<0.001	0.68	<0.001	0.63	<0.001	0.63	<0.001
Other (%)	0.70	<0.001	0.65	<0.001	0.59	0.001	0.73	<0.001

### Land-use exposure and walking

Overall, 60% of study participants reported engaging in some weekly leisure walking, and nearly half reported some walking for transport per week (49.85%). Only age significantly predicted walking for leisure (p = 0.047), while no demographic characteristics were associated with transport walking. Nonetheless, all models exploring which neighbourhood buffer showed relationships between percentage land-use exposure and the two walking behaviours were adjusted for gender, age, education, marital status, BMI, physical functioning, and clustering. Table 6 reports the adjusted odds ratios for the standard buffers and Table 7 the new buffers. Significant relationships were found for percentage exposure to *recreational and park*, *industrial*, and *residential* land-uses and some walking for leisure in both the circular and variable width buffers. Leisure walking was also significantly associated with percentage exposure to *recreational and park* and *other* land-uses, while *industrial* land-use approached significance in the RIC polygon buffer. The only land-use type related to transport walking was *utilities/communications*, which was found across all buffers with the exception of the RIC ellipse buffer.

**Table 6:** Adjusted odds ratios examining leisure and transport walking by percentage land-use exposure measured with standard buffers (n = 325)

**Leisure walking**	**Circular buffer**			**Polygon Network buffer**			**Line-based network buffer**		
	**OR**	**CI**	**p**	**OR**	**CI**	**p**	**OR**	**CI**	**p**
Commercial (%)	1.01	0.98-1.03	0.541	1.03	0.99-1.06	0.136	1.03	0.99-1.08	0.168
Institutional (%)	1.03	0.98-1.09	0.248	1.04	0.99-1.08	0.091	1.08	0.99-1.16	0.054
Recreational and park (%)	1.02	1.00-1.04	0.027	1.04	0.98-1.11	0.186	1.05	0.97-1.14	0.252
Industrial (%)	0.88	0.79-0.98	0.023	0.78	0.61-1.00	0.051	0.79	0.58-1.07	0.126
Residential (%)	0.98	0.97-0.99	0.039	0.99	0.98-1.01	0.562	0.99	0.97-1.01	0.532
Utilities/Communications (%)	0.83	0.64-1.08	0.171	0.95	0.84-1.07	0.411	1.00	0.85-1.16	0.959
Other (%)	1.00	0.99-1.02	0.892	1.00	0.98-1.01	0.623	1.00	0.98-1.02	0.834
**Transport walking**									
Commercial (%)	0.98	0.94-1.03	0.511	1.03	0.96-1.11	0.366	1.04	0.96-1.13	0.344
Institutional (%)	1.06	0.97-1.16	0.201	1.00	0.96-1.05	0.924	1.03	0.94-1.13	0.484
Recreational and park (%)	1.00	0.98-1.02	0.853	1.00	0.93-1.08	0.963	0.99	0.90-1.08	0.776
Industrial (%)	0.98	0.78-1.22	0.843	0.75	0.53-1.06	0.103	0.70	0.47-1.04	0.079
Residential (%)	1.01	0.98-1.04	0.521	0.99	0.97-1.02	0.489	0.99	0.97-1.02	0.473
Utilities/Communications (%)	0.72	0.55-0.94	0.016	0.85	0.74-0.99	0.037	0.83	0.72-0.97	0.021
Other (%)	0.99	0.97-1.01	0.516	1.00	0.98-1.02	0.674	1.01	0.98-1.03	0.607

**Table 7:** Adjusted odds ratios examining leisure and transport walking by percentage land-use exposure measured with new buffers (n = 325)

**Leisure walking**	**Variable width buffer**			**RIC polygon buffer**			**RIC ellipse buffer**			**RIC line buffer**		
	**OR**	**CI**	**p**	**OR**	**CI**	**p**	**OR**	**CI**	**p**	**OR**	**CI**	**p**
Commercial (%)	1.01	0.98-1.05	0.398	1.01	0.99-1.04	0.250	1.01	0.99-1.02	0.440	1.02	0.99-1.04	0.160
Institutional (%)	1.03	0.98-1.07	0.210	1.04	0.99-1.08	0.063	1.00	0.96-1.04	0.930	1.02	0.98-1.06	0.301
Recreational and park (%)	1.03	1.01-1.05	0.007	1.03	1.02-1.05	<0.001	1.02	0.99-1.05	0.068	1.03	0.99-1.08	0.141
Industrial (%)	0.81	0.67-0.98	0.030	0.94	0.89-1.00	0.052	0.96	0.91-1.02	0.168	0.89	0.78-1.01	0.075
Residential (%)	0.98	0.97-0.99	0.039	0.99	0.97-1.01	0.220	0.99	0.97-1.01	0.497	0.99	0.97-1.01	0.385
Utilities/Communications (%)	0.87	0.71-1.07	0.201	0.98	0.90-1.06	0.596	0.99	0.90-1.08	0.776	1.00	0.97-1.03	0.953
Other (%)	1.00	0.99-1.02	0.839	0.98	0.96-0.99	0.024	0.98	0.96-1.00	0.100	0.99	0.97-1.01	0.297
**Transport walking**												
Commercial (%)	0.99	0.93-1.06	0.753	1.03	0.99-1.07	0.200	1.01	0.99-1.04	0.278	1.03	0.99-1.06	0.104
Institutional (%)	1.04	0.96-1.12	0.353	1.01	0.95-1.08	0.657	0.98	0.93-1.04	0.537	1.00	0.93-1.07	0.937
Recreational and park (%)	1.00	0.98-1.02	0.963	1.00	0.98-1.02	0.897	1.01	0.98-1.03	0.549	1.00	0.95-1.04	0.846
Industrial (%)	0.98	0.68-1.44	0.939	0.96	0.87-1.07	0.502	0.98	0.88-1.08	0.662	0.89	0.75-1.06	0.201
Residential (%)	1.01	0.98-1.04	0.668	1.00	0.97-1.03	0.882	0.99	0.97-1.02	0.592	0.99	0.96-1.01	0.348
Utilities/Communications (%)	0.77	0.63-0.93	0.009	0.84	0.75-0.94	0.003	0.87	0.75-1.04	0.152	0.93	0.89-0.96	<0.001
Other (%)	0.99	0.98-1.01	0.639	0.99	0.97-1.01	0.336	0.99	0.96-1.01	0.302	1.00	0.98-1.02	0.847

As an indication of model goodness of fit, the Corrected Quasi likelihood under Independence model Criterion (QICC) values for all models are presented in Table 8. Smaller values indicate better goodness of fit. Models using the RIC polygon and RIC ellipse buffers consistently show better goodness of fit values, followed by the RIC line buffer. The circular, polygon network, line-based network, and variable width buffers all show similar model fit values.

**Table 8:** Corrected Quasi Likelihood under Independence Model Criterion (QICC) values for models examining leisure and transport walking by percentage land-use exposure for each buffer (n = 325)

**Leisure walking**	**Circular buffer**	**Polygon Network buffer**	**Line-based network buffer**	**Variable width buffer**	**RIC polygon buffer**	**RIC ellipse buffer**	**RIC line buffer**
Commercial (%)	432.12	430.80	430.96	431.90	411.63	412.06	419.58
Institutional (%)	431.25	430.50	429.43	431.41	410.36	412.78	421.16
Recreational and park (%)	429.70	430.91	431.13	429.20	407.52	409.55	419.42
Industrial (%)	431.14	431.32	431.53	431.05	411.97	412.28	420.95
Residential (%)	429.49	431.91	431.81	429.44	411.22	412.16	420.74
Utilities/Communications (%)	430.99	431.93	432.33	431.21	412.74	412.77	421.74
Other (%)	432.32	432.02	432.28	432.30	409.29	410.29	420.73
**Transport walking**							
Commercial (%)	441.64	442.82	442.90	442.94	417.76	418.42	427.07
Institutional (%)	439.59	443.82	442.84	441.49	420.33	420.90	431.57
Recreational and park (%)	443.85	443.90	443.60	443.88	421.34	420.81	431.59
Industrial (%)	443.98	442.85	442.38	443.92	421.56	421.55	430.87
Residential (%)	441.72	442.08	441.80	442.98	421.28	419.86	427.32
Utilities/Communications (%)	439.22	440.13	440.46	439.21	419.70	421.49	428.68
Other (%)	443.06	443.16	442.75	443.44	419.98	420.77	431.17

## Discussion

### Summary results and methodologies

To explore the operationalization of neighbourhood, our first purpose was to develop alternative neighbourhood buffers that more accurately defined individual spatial exposure. Table 9 summarizes the standard buffers and four new buffering approaches, identifies the underlying data needs (for creation), and highlights the pros and cons of each. To our knowledge, this is one of the few studies to distinguish and test a range of buffers not previously used in built environment and health research [21,30]. While others have identified the need to re-examine neighbourhood buffers, and remind us that additional options do exist (standard deviation ellipses, convex hulls and kernel densities), they also note that additional information concerning how individuals spatially interact with their environment is required [28,30]. In this study, GPS technology provided accurate representations of human-environment interactions for this purpose.

**Table 9:** Summary of new and standard buffers

**Buffer**	**Short description**	**Based on**	**Pros**	**Cons**
Circular	Area ‘as the crow flies’ delineated as a predefined distance from a reference point	Radius from central location	Easy to create	May incorporate areas not used for physical activity
Polygon Network	Area within a polygon up to a certain distance from a central location when travelling along a network	Road network	More accurately represents area activity can take place	May incorporate areas not used for physical activity,
Line-based network	Land of a predefined distance either side of a road up to a certain distance from a central location when travelling along a network	Road network	Only represents land along a network	May under represent areas used for physical activity
Variable width	Land as defined by circular and/or network buffer limited by cost of travel from a central location	Cost of movement across a grid cell	Takes actual walking behaviour into account	Requires information on walking behaviour
RIC polygon	Area identified as the smallest convex hull around all recreational, institutional, commercial (RIC) facilities and a central location	Facility locations	Captures neighbourhood orientation, only land between a central location and up to possible destinations is included, fast polygon generation	Inaccessible land included
RIC ellipse	All land within an ellipse created around recreational, institutional, and commercial (RIC) facilities within a predefined distance from a reference point	Facility locations	Captures neighbourhood orientation, shows directional information	Possibly excludes the home from the buffer, inaccessible land included
RIC line	Land of a predefined distance either side of a road from a central location to recreational, institutional, and commercial (RIC) facilities along a network	Road network, facility locations	Captures neighbourhood orientation, includes only land between a central location and facilities people are expose to	Requires information on walking behaviour and only incorporates shortest routes to facilities

By exploring the differences between standard buffering techniques and GPS informed buffering approaches, we help to build the bridge between individual built environment exposures and the spatial extend for which environmental measures are derived. Results from our second study show that when we reorientate the ‘neighbourhood’ based on GPS tracking data and spatial phenomena participants were exposed to, measures of land-use exposure vary, a result demonstrated by Zenk at al., [30]. This was especially true for *residential* and *institutional* land-uses, although it should be noted that these land-use classes influenced the spatial orientation of the RIC buffers. Furthermore, the strength of correlations between traditional and new buffers varied for land-use exposure percentage. Whilst these results by no means highlight the most appropriate buffer delineation technique and orientation for measuring land-use exposure, they emphasize the fact that land-use exposure changes when empirical measures of behaviour are used to inform buffer choice.

Thirdly, we examined associations between land-use exposure and walking across each neighbourhood buffer. We found few significant relationships between land-use exposure and walking for either leisure or transport purposes, with the only exceptions being some land-use exposures being associated with walking for leisure in the circular and variable width buffers. Although few significant results were identified, the model fit statistics indicated that land-use exposure for the new RIC buffers provided a better fit for walking than the circular, variable width, or polygon network based buffers. This shows that built environment measures based on where individuals actually walk, may be more suitable when attempting to investigate built environment correlates of walking.

Our findings have a number of implications for the way research is conducted in the field. Most studies rely on neighbourhood buffers from which measures of the built environment are then derived [see 1]. Whilst some simply vary buffer size, there has been limited progress in understanding the spatial extent of healthy behaviours, in order to select the most appropriate space in which to measure environmental influences that relate to behaviour within that space. That is to say, behaviour, in this case walking, may or may not occur within a predefined buffer [29,30]. Discussions concerning the validity of the ‘buffer’ as a neighbourhood definition have intensified, with some calling for place to be identified in terms of time, space and scale [27-29,52]. As Cummin’s et al. [52] argues, notions of place vary with time and space, and understanding the scale of environmental influences on health is critical for moving place-based empirical health research beyond its current state. We have shown that GPS technology can be used to assist researchers in measuring the human environment relationship across space. Though inconclusive, our findings highlight that we can expand our understanding concerning the most appropriate neighbourhood measures from which to examine built environment correlates of walking, and further research with larger samples, using different buffer techniques, is needed.

### Strengths/limitations

The use of objective movement data from GPS devices was a strength of the study and found to be appropriate for analysis of this kind. Using GPS data made it possible to observe travel patterns, without the participant burden and recall bias issues related to travel logs or diaries. However, the use of GPS technology in Health research is still fairly new and there are several issues to consider in terms of data collection, data accuracy, behaviour classification, and analyses.

Technically speaking, physical and built features such as dense tree canopies, topography, tall buildings (urban canyons), and transportation tunnels can result in signal loss and multipath errors [34,43,53]. Operationally, the time taken for a GPS to communicate with satellites and calculate a geographic position is impacted by cold starts where short trips can go unrecorded [53]. Similarly, quick trips between buildings can go undetected if a GPS has been turned on but blocked from acquiring a satellite signals.

On the ground, technological advancements such as dead reckoning, increased sensitivity of receiver antennas, positional augmentation using coordinates collected by a mobile phone [53], mobile phone triangulation using WiFi hotspots and mobile phone towers, and radio frequency identification tags [21] provide options for augmenting signal loss. Furthermore, access to new positional satellites such as Europe’s GALILLEO system promise to increase positional accuracy and decrease signal loss in urban environments through greater satellite coverage the world over [54].

Whilst data management in GPS studies is a critical issue because of the massive quantities of data produced by interval recordings [53] we found the prevalence of errors associated with defining the start and end points of trips more problematic. In order to make sense of the huge amounts of data, appropriate platforms such as PALMS are needed to identify and classify trips in large strings of data points. However, research tools like PALMS are only as good as the underlying rules used to define and classify trips, and in some cases human intervention may still be required.

Our use of PALMS was during the developmental stages of the system therefore visual examinations were used to validate trip classifications. Misclassification of trips included stationary trips classified as vehicle, bicycle and walking, mixed tips classified as a single type and vice versa, and one type of trip classified as another. Distinguishing between walking and use of electric mobility scooters proved difficult in our cohort of older adults as their speeds of movement can be similar. The most recent version of PALMS allows the user to specify thresholds however in our case we identified thresholds through trial and error relaying our new parameter requirements to the PALMS staff. Therefore, further research is needed to overcome problems in data treatment in order to make sense of large and highly accurate data, an issue PALMS is continuing to address.

As several studies have identified, the elderly tend to walk more frequently in their neighbourhood than younger people [31,50,53] and our older adults sample, albeit small, reflected the spatial walking patterns of the participants. But the buffer distances that were found best for seniors may not be appropriate for non-elderly populations, as they may be longer for younger persons [51]. Furthermore, the types of land-uses that older adults are exposed to when walking may be different than those for other age groups. Our findings are limited in their generalizability to other age groups but also in terms of geography as well, as results may vary in different urban and rural locations. Therefore, further research across the population and in varying geographic contexts is needed, as age and geographic specific buffering approaches may be required to best measure built environment influences on walking.

## Conclusion

In conclusion, this pilot study highlights the possibilities that exist for re-examining the spatial extent from which built environment measures are derived in health related research. The use of GPS technology allowed for an objective view of how humans interact with their environment across space, and provided a tool to challenge the way in which neighbourhoods are defined and operationalized. Spatially informed buffers specific to the behaviour in question showed better model fit and influenced relationships between built environment measures and walking for leisure among older adults. Future research should capitalise on the potential of GPS technology to explore (and be creative in) how we define neighbourhoods in research studies across a wider range of population age groups and types of healthy behaviours.

## Competing interests

The authors declare that they have no competing interests.

## Authors’ contributions

BJB conceived of the project, oversaw GIS analysis and prepared much of the manuscript. AN collected the GPS data, provided statistical analysis and contributed to the manuscript development and editing. SN provided GIS analysis, contributed to the development of new GIS methods and early drafts of the manuscript. All authors have read and approved the final manuscript.
